# Conspiracy Beliefs, Rejection of Vaccination, and Support for hydroxychloroquine: A Conceptual Replication-Extension in the COVID-19 Pandemic Context

**DOI:** 10.3389/fpsyg.2020.565128

**Published:** 2020-09-18

**Authors:** Paul Bertin, Kenzo Nera, Sylvain Delouvée

**Affiliations:** ^1^LAPCOS, Université Côte d’Azur, Nice, France; ^2^Center for Social and Cultural Psychology, Université Libre de Bruxelles, Brussels, Belgium; ^3^Fonds de la Recherche Scientifique (FRS), Bruxelles, Belgium; ^4^EA1285 Laboratoire de Psychologie, Cognition, Comportement, Communication (LP3C), University of Rennes, Rennes, France

**Keywords:** vaccination, chloroquine, conspiracy beliefs, conspiracy mentality, attitude toward science, pandemic (COVID-19), COVID–19, vaccination intention

## Abstract

Many conspiracy theories appeared along with the COVID-19 pandemic. Since it is documented that conspiracy theories negatively affect vaccination intentions, these beliefs might become a crucial matter in the near future. We conducted two cross-sectional studies examining the relationship between COVID-19 conspiracy beliefs, vaccine attitudes, and the intention to be vaccinated against COVID-19 when a vaccine becomes available. We also examined how these beliefs predicted support for a controversial medical treatment, namely, chloroquine. In an exploratory study 1 (*N* = 409), two subdimensions of COVID-19 conspiracy beliefs were associated with negative attitudes toward vaccine science. These results were partly replicated and extended in a pre-registered study 2 (*N* = 396). Moreover, we found that COVID-19 conspiracy beliefs (among which, conspiracy beliefs about chloroquine), as well as a conspiracy mentality (i.e., predisposition to believe in conspiracy theories) negatively predicted participants’ intentions to be vaccinated against COVID-19 in the future. Lastly, conspiracy beliefs predicted support for chloroquine as a treatment for COVID-19. Interestingly, none of the conspiracy beliefs referred to the dangers of the vaccines. Implications for the pandemic and potential responses are discussed.

## Introduction

Conspiracy theories can be defined as “attempts to explain the ultimate causes of significant social and political events and circumstances with claims of secret plots by two or more powerful actors” (Douglas et al., 2019, p. 4). These beliefs tend to appear in social crisis situations, which are times of heightened collective uncertainty and fear (van Prooijen and Douglas, 2017). It has been proposed that these beliefs are a response to psychological needs (Douglas et al., 2017), and might constitute attempts to understand complex, otherwise hardly understandable and predictable threatening situations (Franks et al., 2013). Hence, it is not surprising that conspiracy beliefs have flourished with the COVID-19 pandemic, and that medical misinformation spreads at a spectacular rate (Kouzy et al., 2020). Interestingly, conspiracy beliefs also surged during the 1918–1919 Spanish flu pandemic (Spinney, 2017) and the 2009 H1N1 outbreak (Bangerter et al., 2012).

Conspiracy beliefs may also influence the course of a crisis that initially favored their appearance. Indeed, conspiracy beliefs have consequences, notably in the health domain (van Prooijen and van Douglas, 2018). For example, exposure to anti-vaccine conspiracy theories decreases vaccination intention (Jolley and Douglas, 2014). This relation is not limited to conspiracy theories about vaccines, as authors have found that the endorsement of “classic” conspiracy beliefs unrelated to vaccination (e.g., about JFK, the Moon Landing) is also associated with negative attitudes toward vaccines (Lewandowsky et al., 2013). This might be explained by the fact that the endorsement of some conspiracy beliefs is a powerful predictor of the endorsement of others, even when they are seemingly unrelated (e.g., Goertzel, 1994; Swami et al., 2011). As a result, it has been proposed that conspiracy beliefs are associated with a generic belief system, which has been given names such as “monological belief system” (Goertzel, 1994), or “conspiracy mentality” (Moscovici, 1987). Overall, there might be a negative relation between conspiracy beliefs and attitude toward scientific medicine, Lamberty and Imhoff (2018) have shown that conspiracy mentality was associated with a preference for alternative medicines over evidence based, biomedical treatments.

In this research, we sought to replicate the aforementioned relationship between conspiracy beliefs, rejection of vaccination, and support for alternative treatments, in the context of the COVID-19 pandemic. Indeed, previous studies were conducted before the sanitary crisis that the world is currently experiencing. Replicating past results is essential for at least two reasons. Firstly, replication is necessary to establish the validity of frequentist statistical inferences (Krueger, 2001), which is the overwhelmingly dominant statistical approach in the psychological literature (Blanca et al., 2018). Secondly, given that research in psychology is in a post-replication crisis era (Anvari and Lakens, 2018), advice from psychological science must be taken with great caution, especially in a situation such as the ongoing COVID-19 pandemic (IJzerman et al., 2020). In this context, replication studies might strengthen social psychological knowledge (Rosenfeld et al., 2020) and constitute a safety baseline needed to build evidence-based policies and effective sanitary guidelines.

Replication efforts should be encouraged even more given the magnitude of the stakes. In the context of the COVID-19 pandemic, conspiracy beliefs may foster distrust toward health authorities and their recommendations, which could potentially impede efforts to put an end to the pandemic. In the short term, respect for containment behavior guidelines (e.g., social distancing) is crucial to limit the spread of the pandemic we are currently experiencing, because the development of a treatment (including a vaccine) could take months (World Health Organization, 2020). However, in the long run, the development and distribution of a vaccine against COVID-19 might be a necessary step to put an end to the pandemic (Le et al., 2020).

In this research, we examine how the endorsement of various COVID-19 conspiracy beliefs is predictive of two vaccine-related outcomes: attitude toward vaccination science (Studies 1 and 2), conceptually replicating a research by Lewandowsky et al. (2013), and intention to be vaccinated against COVID-19 when a vaccine becomes available (Study 2), conceptually replicating Jolley and Douglas (2014). In Study 2, we also examined the extent to which COVID-19 conspiracy beliefs are associated with positive attitudes toward a controversial COVID-19 treatment, namely, chloroquine.

Note that in both studies, we referred to COVID-19 (the disease) and not to SARS-CoV-2 (the virus that causes the disease), to be in line with the terminology of the French media coverage of the pandemic and therefore avoid misunderstandings.

## Study 1

In Study 1, our goal was to explore the relationship between COVID-19 conspiracy beliefs and attitudes toward vaccines science. Data, materials in French (with English translation) and analyses are available on the OSF repository at the following address: https://osf.io/3qyf4/?view_only=c2aa291fb1604b73aef9057cfc41980e.

### Method

#### Sampling and Procedure

The online questionnaire was disseminated by the authors on Facebook, Twitter, and Linkedin from March 19 (i.e., 2 days after the official beginning of the lockdown in France) to March 27. In total, 609 participants participated in the survey. Two hundred participants were removed from the data for not completing the questionnaire, for failing the attention or seriousness checks, or for being under 18 years old. The final sample was constituted of 409 participant (299 women and 3 “other,” *Mage* = 28.4, *SD* = 11.4, *min* = 18, *max* = 72, see the Supplementary Material for the geographical localization of participants), which is above the threshold of *N* = 250 requested to achieve correlations stability (Schönbrodt and Perugini, 2013). For a given power of 0.90, this sample size enabled us to detect correlations of *r* = 0.16 with two tailed tests.

#### Materials

For each scale, participants were asked to give their response on a 5-point scale ranging from Strongly Disagree (coded 1) to Strongly Agree (coded 5).

##### COVID-19 Conspiracy beliefs

Nine items were designed to capture the endorsement of some COVID-19 conspiracy theories currently popular in France (Conspiracy Watch, 2020). Given the wide variety of COVID-19 conspiracy theories (Van Bavel et al., 2020), we designed items tapping into three group-based categories: conspiracy theories involving a threatening foreign outgroup, namely, China (three items, e.g., “COVID-19 is a bacteriological weapon used by the Chinese Communist Party to create panic in the West”), conspiracy theories involving unspecified outgroups (i.e., not referring to any foreign country outgroups, three items, e.g., “Industrials will use the coronavirus pandemic to justify higher prices and make a profit”), and conspiracy theories involving members of the national ingroup, namely, the French government (three items, e.g., “The French government uses the current pandemic to keep significant reforms and challenges quiet”). Some authors have emphasized the theoretical and empirical relevance of distinguishing between national ingroup and outgroup conspiracy beliefs (e.g., Cichocka et al., 2016). Exploratory factor analysis with Oblimin rotation revealed a two factor structure yielding a satisfactory fit, one consisting of the three “foreign outgroups” and two “unspecified outgroups” items (α = 0.88), and one combining the three “ingroup” items and one “unspecified outgroup” (α = 0.77). The two factors were substantially correlated, *r* = 0.53, *p* < 0.001 (see Table 2 for additional analyses for the item loadings). The dimensions were labeled “outgroup conspiracy beliefs” and “ingroup conspiracy beliefs,” respectively.

##### Attitude toward vaccination

We translated into French the 5-items scale (1 reverse coded) developed by Lewandowsky et al. (2013). We used three items due to length restrictions (e.g., “I believe that vaccines are a safe and reliable way to help avert the spread of preventable diseases”, α = 0.83).

##### Sociodemographic measures

Participants reported their age, gender (M/F/Other), geographic location, and political orientation on a scale ranging from 1 (far left) to 9 (far right), with the possibility to tick “other.”

### Results and Discussion

Descriptive statistics and correlations between measured variables are displayed in Table 1. We carried out hierarchical regression analyses to examine whether the two dimensions of COVID-19 conspiracy beliefs predicted attitudes toward vaccination, controlling for gender, age, and political orientation at step 1 (see Table 2). Since the two factors of COVID-19 conspiracy beliefs were substantially correlated both to each other and to attitudes toward vaccines, they were first tested as predictors in separate regressions. In the two models, attitudes toward vaccines were negatively predicted by both “outgroup” conspiracy beliefs, β = −0.052, *95% CI*[−0.61, −0.42], *t* = −10.36, *p* < 0.001, and “ingroup” conspiracy beliefs, β = −0.44, *95% CI*[−0.53, −0.34], *t* = −8.71, *p* < 0.001. Finally, we tested a model integrating both dimensions as predictors, with outgroup COVID-19 conspiracy beliefs introduced at step 2, and ingroup COVID-19 conspiracy beliefs introduced at step 3 (see Table 2). The relationship remained significant for both the “ingroup” factor, β = −0.23, *95% CI*[−0.34, −0.12], *t* = −4.19, *p* < 0.001, and the “outgroups” factor, β = −0.38, *95% CI*[−0.50, −0.27], *t* = −6.64, *p* < 0.001, Δ*R*^2^ = 0.03, *p* < 0.001. The fact that confidence intervals for the standardized coefficients do not overlap suggests that the “outgroups” factor might be more strongly associated with the dependent variable than the “ingroup” factor.

**TABLE 1:** Correlations, means, and standard deviations for measured variables (study 1).

	*Mean*	*SD*	1	2	3
1. Outgroup COVID-19 conspiracy beliefs	1.44	0.69	–		
2. Ingroup COVID-19 conspiracy beliefs	2.47	0.97	0.53***	–	
3. Attitude toward vaccination	3.37	0.47	−0.23***	−0.28***	–
4. Political orientation	4.19	1.94	0.26***	0.04	0.06

***p < 0.001, *N* = 409 except for political orientation (*N* = 314). All variables were measured using a 5-point Likert scale, except for political orientation (9 points).

**TABLE 2:** Hierarchical regressions on attitude toward vaccination (study 1).

Independent variables	Dependent variables					
	Attitude toward vaccination					
	*B*	95% CI	*t*	*p*	Total *R*^2^	Δ *R*^2^
**Step 1**					0.04	
Gender	−0.17	[−0.28, −0.06]	−3.08	0.01		
Age	0.02	[−0.08, 0.13]	0.50	0.61		
Political orientation	−0.12	[−0.023, −0.01]	−2.31	0.02		
**Step 2 (outgroup conspiracy beliefs)**					0.29	0.24
Gender	−0.10	[−0.19, −0.01]	−2.09	0.03		
Age	0.01	[−0.08, 0.10]	0.26	0.78		
Political orientation	0.01	[−0.08, 0.11]	0.26	0.78		
Outgroup COVID-19 conspiracy beliefs	−0.52	[−0.61, −0.42]	−10.36	<0.001		
**Step 2 (ingroup conspiracy beliefs)**					0.23	0.18
Gender	−0.10	[−0.20, −0.01]	−2.09	0.04		
Age	0.01	[−0.08, 0.11]	0.28	0.77		
Political orientation	−0.10	[−0.20, −0.01]	−2.15	0.03		
Ingroup COVID-19 conspiracy beliefs	−0.44	[−0.53, −0.34]	−8.71	<0.001		
**Step 3**					0.33	0.03
Gender	−0.08	[−0.17, 0.01]	−1.77	0.07		
Age	0.01	[−0.08, 0.10]	0.19	0.84		
Political orientation	−0.01	[−0.01, 0.08]	−0.23	0.81		
Outgroup COVID-19 conspiracy beliefs	−0.38	[−0.50, −0.27]	−6.64	<0.001		
Ingroup COVID-19 conspiracy beliefs	−0.23	[−0.34, −0.12]	−4.19	<0.001		

**N* = 409.*

Hence, regardless of their specific content, the more participants endorsed COVID-19 conspiracy beliefs, the less likely it was that they held a positive attitude toward vaccination. This result is congruent with past research showing that conspiracy beliefs are related to negative attitudes toward vaccination (Lewandowsky et al., 2013).

## Study 2

We designed a second study to replicate and strengthen results from study 1. To grasp a more comprehensive understanding of the relationship between COVID-19 conspiracy beliefs and vaccination in the context of the pandemic, we examined if conspiracy beliefs were also negatively associated with the intention to be vaccinated against the disease (a relationship previously reported in Jolley and Douglas, 2014). For the same reason, we included a measure of conspiracy mentality, that is, the general propensity to subscribe to theories blaming a conspiracy of ill-intending individuals or groups for important societal phenomena (Bruder et al., 2013), as an additional independent variable. As we mentioned in the introduction, previous studies found conspiracy mentality to be related to negative attitudes toward vaccination (Lewandowsky et al., 2013).

Lastly, we wanted to examine the extent to which COVID-19 conspiracy beliefs would predict support for a controversial treatment against disease, namely, chloroquine. Chloroquine is a well-known anti-malarial drug that has been mostly promoted by the French infectious disease expert Didier Raoult. In April, a poll reported that 59% of a representative sample of the French population believes this treatment to be effective (Institut français d’opinion publique [IFOP], 2020). Lamberty and Imhoff (2018) have shown that conspiracy mentality is associated with a preference for alternative therapies over biomedical therapies. In this regard, the situation with chloroquine is interesting, because it is a drug produced by pharmaceutical companies, that is promoted by a prominent medical researcher. Hence, one could expect conspiracy theories to be negatively related with trust in this treatment. However, many chloroquine advocates appear to mobilize conspiracy theories to defend this treatment, arguing that pharmaceutical companies are willing to discredit it because generalizing it would jeopardize potential profits. We therefore expected that despite the fact that it is a medication produced by pharmaceutical companies, COVID-19 conspiracy beliefs would predict support for chloroquine treatment. Given their prevalence on French social media, we moreover included “pro-chloroquine” conspiracy beliefs among the independent variables.

Whereas Study 1 was exploratory, Study 2 aimed at testing a set of pre-registered hypotheses^1^. We hypothesized that COVID-19 conspiracy beliefs (ingroup, outgroup, and pro-chloroquine) would be (1) negative predictors of both pro-vaccination attitudes and vaccination intention, and (2) positive predictors of pro-chloroquine attitudes. Lastly, we included conspiracy mentality as an exploratory measure.

### Method

#### Sampling and Procedure

The study was disseminated online among undergraduate students from Rennes 2 and Lille Universities who were awarded course credit for answering. It was also shared by authors on social media in order to diversify the sample, from April 17 to April 25. In total, 469 participants participated in the study, out of which 396 remained (280 women and 6 “other,” *Mage* = 26.1, *SD* = 10.3, *min* = 18, *max* = 70, see the Supplementary Material for information about participants’ level of education) after excluding participants who did not comply to the inclusion criteria (see pre-registration). For a given power of 0.90, the sample size enabled us to detect correlations of *r* = 0.16 with two tailed tests.

#### Measures

Unless otherwise indicated, participants answered on a 5-point scale ranging from Strongly Disagree (coded 1) to Strongly Agree (coded 5). Measures of attitudes and conspiracy beliefs about chloroquine, as well as vaccination intention, were pretested for internal reliability and ceiling and floor effects in an online preliminary study (*N* = 81, see Supplementary Material in the OSF repository for further details).

##### COVID-19 conspiracy beliefs

We used the same scale as in Study 1, and added a conspiracy theory about the creation of the coronavirus by a famous French laboratory (“Coronavirus has been created and patented by the Pasteur Institute in the early 2000s”). The two factors structure found in Study 1 yielded a satisfactory fit (CFI = 0.94, TLI = 0.93, RMSEA = 0.08), and the dimensions returned satisfactory internal reliability (α = 0.76 and 0.87 for, respectively, “ingroup” and “outgroups” factors, see Supplementary Table 4). It is worth noting that the new item about the Pasteur Institute was loaded onto the “outgroup” dimension, along with other conspiracy theories involving scientists and foreign governments. One explanation could be related to the magnitude of the considered conspiracies. Whereas the ingroup conspiracy theories have consequences at the scale of the nation (e.g., municipal elections, political reforms), the outgroup conspiracy theories have potentially worldwide consequences, with the Pasteur Institute conspiracy falling in this latter group.

##### Chloroquine conspiracy beliefs

We designed a 6-item scale to assess participant beliefs in popular “pro-chloroquine” conspiracy theories (e.g., “Pharmaceutical industries, together with the government, avoid chloroquine based treatment diffusion to protect its financial interests,” α = 0.88). The confirmatory factor analysis of the scale yielded an acceptable fit for a single factor structure, suggesting that the items captured a single construct (CFI = 0.97, TLI = 0.95, RMSEA = 0.10). Note that when carrying out an exploratory factor analysis on ingroup, outgroup, and chloroquine conspiracy beliefs altogether, the three postulated dimensions were observed (see Supplementary Table 4).

##### Conspiracy mentality questionnaire (CMQ)

The general propensity to endorse conspiracy theories was measured with a validated French translation of the Conspiracy Mentality Questionnaire (CMQ; Bruder et al., 2013, translation by Lantian et al., 2016). It is a 5-item measure designed to assess an individual’s tendency to engage in general conspiracist ideation (e.g., “I think that many very important things happen in the world, which the public is never informed about”, α = 0.84). Participants rated how true they thought a given item was on an 11-point scale (from 0% = “Certainly not” to 100% = “Certain”).

##### Attitude toward chloroquine treatment

We created a 5-item scale (2 reverse-coded) to measure participants’ attitudes toward chloroquine medical treatment for COVID-19 (e.g., “This treatment is to date the most effective one against COVID-19”, α = 0.88). Items were preceded by a paragraph introducing the question of chloroquine (“We hear a lot about the potential of a drug, chloroquine, to cure COVID-19 […] what is your opinion on the topic?”). The confirmatory factor analysis of the scale yielded a satisfactory fit for a single factor structure (CFI = 0.98, TLI = 0.96, RMSEA = 0.08).

##### Attitude toward vaccination

We used the full 5-item scale (e.g., “Vaccinations are one of the most significant contributions to public health,” α = 0.84) developed by Lewandowsky et al. (2013).

##### Vaccination intention

We adapted the single item used by Jolley and Douglas (2017), to assess behavioral intention to be vaccinated against COVID-19. We asked participants what they would do if a COVID-19 vaccine were developed and validated by the health authorities, and they had the opportunity to be vaccinated next week. Participants answered on a scale ranging from 1 (“I would definitely not be vaccinated under any circumstances”) to 7 (“I would be vaccinated without any hesitation”).

##### Sociodemographic measures

Participants reported their age, gender (M/F/other), and political orientation on a scale ranging from 1 (far left) to 9 (far right), with the possibility to tick “other.” They also reported their level of education on a multiple choice question (ranging from no diploma to doctoral degree).

### Results

#### Confirmatory Analyses

Descriptive statistics and correlations between measured variables are displayed in Table 3. To test our hypotheses, we carried out hierarchical regression analyses that controlled for age, gender, and political orientation at step 1 (see Table 4). As can be seen in the table, all of our hypotheses were corroborated, as all types of conspiracy beliefs (outgroup, ingroup, pro-chloroquine) were negative predictors of both positive attitudes toward vaccination and intention to get vaccinated for the disease in the future (Step 2). Moreover, also congruent with our expectations, all types of conspiracy beliefs positively predicted a pro-chloroquine attitude. Contrary to study 1, when outgroup COVID-19 conspiracy beliefs were included in the model, ingroup COVID-19 conspiracy beliefs were significantly related to none of the dependent variables (Step 3). This echoes the fact that in Study 1, outgroup COVID-19 conspiracy beliefs were more strongly associated with the dependent variable than ingroup conspiracy beliefs.

**TABLE 3:** Correlations, means, and standard deviations for measured variables (study 2).

	*Mean*	*SD*	1	2	3	4	5	6	7
1. Outgroup COVID-19 conspiracy beliefs	1.60	0.69	–						
2. Ingroup COVID-19 conspiracy beliefs	2.60	0.94	0.41***	–					
3. Chloroquine conspiracy beliefs	2.22	0.89	0.55***	0.50***	–				
4. Attitude toward vaccination	3.86	0.82	−0.41***	−0.25***	−0.54***	–			
5. COVID-19 vaccination intention	4.72	1.80	−0.28***	−0.17***	−0.38***	0.66***	–		
6. Attitude toward chloroquine	2.63	0.76	0.25***	0.18***	0.59***	−0.33***	−0.21***	–	
7. Political orientation	3.52	1.68	–0.02	−0.09^+^	–0.07	0.01	0.04	0.01	–
8. CMQ	6.54	2.13	0.27***	0.30***	0.35***	−0.29***	−0.22***	0.23***	−0.12*

+*p* < 0.10, *p < 0.05, ***p < 0.001. *N* = 396 except for political orientation (*N* = 325). All variables were measured using 5-point Likert scales, except for vaccination intention (7 points), political orientation (9 points), and CMQ (11 points).

**TABLE 4:** Hierarchical regressions on attitude toward vaccination, vaccination intention, and attitude toward chloroquine controlling for gender, age, political orientation (study 2).

Independent variables	Dependent variables																	
	Attitude toward vaccination						Vaccination intention						Attitude toward chloroquine					
	β	95% CI	*t*	*p*	Total *R*^2^	Δ *R*^2^	β	95% CI	*t*	*p*	Total *R*^2^	Δ *R*^2^	β	95% CI	*t*	*p*	Total *R*^2^	Δ *R*^2^
**Step 1**					0.018						0.015						0.017	
Gender	−0.08	[−0.19, 0.02]	−1.58	0.11			−0.10	[−0.21, 0.01]	−1.87	0.06			0.01	[−0.09, 0.12]	0.28	0.77		
Age	0.09	[−0.05, 0.15]	1.06	0.29			0.05	[−0.05, 0.16]	0.92	0.35			0.03	[−0.07, 0.14]	0.68	0.49		
Political orientation	0.01	[−0.09, 0.12]	0.19	0.84			0.04	[−0.06, 0.15]	0.79	0.42			0.01	[−0.09, 0.12]	0.23	0.81		
**Step 2 (outgroup conspiracy beliefs)**					0.16	0.15					0.07	0.05					0.08	0.08
Gender	−0.02	[−0.12, 0.07]	−0.05	0.59			−0.06	[−0.17, 0.04]	−1.23	0.21			−0.02	[−0.13, 0.07]	−0.53	0.59		
Age	0.09	[−0.01, 0.19]	1.92	0.06			0.05	[−0.05, 0.15]	0.94	0.34			0.03	[−0.06, 0.14]	0.71	0.47		
Political orientation	−0.07	[−0.07, 0.12]	0.44	0.65			0.05	[−0.05, 0.15]	0.94	0.34			0.01	[−0.10, 0.11]	0.07	0.93		
Outgroup COVID-19 conspiracy beliefs	−0.39	[−0.49, −0.29]	−7.58	<0.001			−0.23	[−0.34, −0.12]	−4.33	<0.001			0.29	[0.18, 0.40]	5.41	<0.001		
**Step 2 (ingroup conspiracy beliefs)**					0.06	0.04					0.02	0.01					0.04	0.03
Gender	−0.05	[−0.16, 0.05]	−1.05	0.29			−0.08	[−0.19, 0.02]	−1.59	0.11			−0.01	[−0.12, 0.09]	−0.20	0.83		
Age	0.09	[−0.01, 0.19]	1.69	0.09			0.04	[−0.06, 0.15]	0.86	0.38			0.04	[−0.06, 0.15]	0.80	0.42		
Political orientation	−0.01	[−0.11, 0.09]	−0.19	0.84			0.03	[−0.07, 0.14]	0.59	0.54			0.03	[−0.07, 0.14]	0.58	0.55		
Ingroup COVID-19 conspiracy beliefs	−0.22	[−0.32, −0.11]	−3.99	<0.001			−0.11	[−0.22, −0.01]	−1.97	0.05			0.19	[0.08, 0.30]	3.57	<0.001		
**Step 3**					0.17	0.01					0.07	0.01					0.09	0.01
Gender	−0.03	[−0.12, 0.08]	−0.40	0.33			−0.06	[−0.17, 0.04]	−1.19	0.23			−0.03	[−0.14, 0.06]	−0.6	0.49		
Age	0.01	[−0.01, 0.19]	1.87	0.19			0.05	[−0.05, 0.15]	0.93	0.35			0.04	[−0.06, 0.14]	0.7	0.44		
Political orientation	0.01	[−0.08, 0.11]	0.27	0.78			0.04	[−0.05, 0.15]	0.89	0.37			0.01	[−0.09, 0.12]	0.2	0.78		
Outgroup COVID-19 conspiracy beliefs	−0.43	[−0.47, −0.25]	−6.46	<0.001			−0.22	[−0.34, −0.11]	−3.84	<0.001			0.25	[0.14, 0.37]	4.35	<0.001		
Ingroup COVID-19 conspiracy beliefs	−0.06	[−0.19, 0.03]	−1.41	0.15			−0.02	[−0.13, 0.09]	−0.36	0.71			0.10	[−0.01, 0.21]	1.7	0.09		
**Step 2 (chloroquine conspiracy beliefs)**					0.33	0.31					0.15	0.14					0.39	0.38
Gender	−0.03	[−0.12, 0.05]	−0.77	0.44			−0.06	[−0.17, 0.03]	−1.33	0.18			−0.04	[−0.12, 0.04]	−0.95	0.34		
Age	0.06	[−0.02, 0.15]	1.49	0.13			0.03	[−0.07, 0.13]	0.60	0.54			0.07	[−0.01, 0.15]	1.63	0.10		
Political orientation	−0.03	[−0.12, 0.05]	−0.71	0.47			0.01	[−0.08, 0.11]	0.28	0.77			0.06	[−0.02, 0.14]	1.39	0.16		
Chloroquine conspiracy beliefs	−0.56	[−0.65, −0.47]	−12.30	<0.001			−0.38	[−0.48, −0.27]	−7.35	<0.001			0.62	[0.54, 0.71]	14.21	<0.001		
**Step 2 (CMQ)**					0.09						0.06						0.07	
Gender	−0.08	[−0.19, 0.01]	−1.61	0.11			−0.10	[−0.20, 0.01]	−1.89	0.06			0.01	[−0.09, 0.12]	0.26	0.79		
Age	0.09	[−0.01, 0.19]	1.76	0.29			0.04	[−0.05, 0.15]	0.88	0.37			0.04	[−0.06, 0.14]	0.78	0.43		
Political orientation	−0.02	[−0.13, 0.08]	−0.47	0.84			0.01	[−0.09, 0.12]	0.29	0.76			0.04	[−0.06, 0.15]	0.86	0.39		
CMQ	−0.28	[−0.39, −0.18]	−5.37	<0.001			−0.22	[−0.32, −0.11]	−4.05	<0.001			0.27	[0.16, 0.37]	4.97	<0.001		

**N* = 396, CMQ, conspiracy mentality questionnaire.*

#### Exploratory Analyses

We also tested the extent to which conspiracy mentality, rather than belief in specific conspiracy theories, predicted the three outcomes (see Step 2 (CMQ) in Table 4). Conspiracy mentality had the same relationship as COVID-19 and chloroquine conspiracy beliefs with vaccine attitudes, intention to be vaccinated, and pro-chloroquine attitudes.

We might add that, contrary to our expectations, we did not find a strong ceiling effect for the vaccination intention scale (*M* = 4.72; *SD* = 1.80). Strikingly, 22% of the sample (*N* = 87) answered below the median point (4), and among them 29 (7.3% of the sample) reported that they would “refuse vaccination without hesitation.”

## General Discussion

In our studies, various COVID-19 conspiracy beliefs were substantially and negatively related to both positive attitudes toward vaccination science and intention to be vaccinated against COVID-19 in the future. This relationship was observed for conspiracy beliefs accusing outgroups, conspiracy theories involving the French government, “pro-chloroquine” conspiracy beliefs, and conspiracy mentality.

Furthermore, all types of conspiracy beliefs were positively associated with support for an alternative treatment, namely, chloroquine. This deserves some unpacking. Whereas the relationship between positive attitudes toward alternative treatment and conspiracy mentality has been documented (Imhoff and Lamberty, 2018), chloroquine is produced and distributed by pharmaceutical industries (e.g., Sanofi in France, as Plaquenil), and advocated by the infection diseases specialist Didier Raoult, who is a renowned (although controversial) medical scientist. This might be explained by chloroquine being associated with an anti-establishment discourse targeting, among other actors, pharmaceutical companies. Thus, conspiracy beliefs about the dismissal of this treatment, which is itself manufactured by pharmaceutical companies, might paradoxically have become an indicator of individuals’ prejudice against pharmaceutical companies.

Furthermore, this result also puts in perspective the idea that people scoring high on the conspiracy mentality scale are more prone to support a remedy if it comes from a powerless agent (Imhoff and Lamberty, 2018). While Didier Raoult pretends to be the target of pharmaceutical companies, he is the head of a university hospital in Marseille (IHU Méditerranée) and repeatedly reminds his audience that he is a highly respected scientist, and that he is “the elite” (Le Point, 2020).

In both studies, our results suggest that COVID-19 conspiracy beliefs about outgroups (foreign governments and scientists) have stronger relationships to vaccines science attitudes and vaccination intention than conspiracy beliefs about the ingroup (French government and industries). This might be explained both by the foreign origin of the pandemic (e.g., the role of chinese authorities) and distrust toward multinational pharmaceutical companies (among which, the Pasteur Institute). It would surely be of interest to further investigate factors explaining the difference between ingroup and outgroup conspiracy beliefs.

Rather concerning is the fact that in our sample, more than one participant out of five leaned toward refusal of the hypothetical COVID-19 vaccine, even though it was described as having been approved by the health authorities. This is congruent with data showing that the French population is extremely distrustful of vaccines (Ward et al., 2019). If a COVID-19 vaccine were available, 26% of French people would refuse to be vaccinated according to a longitudinal study of a representative sample (Yamey et al., 2020). This proportion, measured for the fifth time since the start of the COronavirus et CONfinement: Enquête Longitudinale (COCONEL) (2020) survey (27 March), appears to remain very stable. While many variables might influence this overall high rate of distrust toward vaccines (e.g., past sanitary scandals in French history, experience of vaccines side effects), it is likely that conspiracy theories are fueling (and potentially fueled by) such distrust.

Lastly, we wish to emphasize that none of the conspiracy beliefs or conspiracy mentality items referred to the dangers of vaccines. Hence, a wide range of conspiracy beliefs seems to be associated with a distrust of vaccines. This is congruent with the idea that conspiracy beliefs are underpinned by a generic belief system, which is characterized by negative attitudes toward powerful groups (Imhoff and Bruder, 2014). It therefore conceptually replicates research conducted before the pandemic (Lewandowsky et al., 2013; Jolley and Douglas, 2014; Imhoff and Lamberty, 2018) as well as during the pandemic (Goldberg and Richey, 2020).

## Limitations and Conclusion

Our research has limits. Firstly, the cross-sectional design we used does not allow for inference to be drawn regarding causality. Although in line with previous research, we suspect that conspiracy beliefs may fuel negative attitudes toward vaccination (Bogart et al., 2010; Jolley and Douglas, 2014), one could hypothesize a reverse causal path, with distrust toward vaccination leading to conspiracy beliefs (e.g., as a confirmatory strategy). People might indeed reject vaccination for non-conspiracist reasons (e.g., religious reasons) and therefore endorse conspiracy theories that legitimize their view.

Secondly, some unmeasured factors may influence negative attitude toward vaccination and vaccination intention, such as concern about drug companies profiteering from vaccines (Martinez-Berman et al., 2020), distrust toward political parties (Rozbroj et al., 2019), or even individuals’ own vaccination history. As for unmeasured sociodemographic variables such as level of education or income, they seem to be overall unrelated to negative attitudes toward vaccination (Hornsey et al., 2018).

Thirdly, the phrasing of our vaccination intention measure may explain the high level of vaccination hesitancy in our sample. Indeed, people may need more information and guarantees about the success of medical trials and possible side-effects before stating their intention to use such a new vaccine.

Lastly, our samples were not representative of the French population, with an overrepresentation of female, southern-located, educated, and left-wing participants. Moreover, online surveys do not reach the population that has no access to the internet (in France, with about 15% of the population have no access to the internet, Institut National de la Statistique et des Etudes Economiques [INSEE], 2019). However, according to a recent poll conducted on a French representative sample, conspiracy beliefs are more endorsed among men and right-wing individuals (Conspiracy Watch, 2019). Thus, we can expect that the results of the present studies might not be overestimated due to unrepresentative sampling. Further research is, however, needed to assess the generalizability of our results to similar yet different contexts (e.g., European and other Western countries).

What should be done in response to the questions investigated in this research? Previous works have shown several ways to reduce the detrimental consequences of conspiracy beliefs. Firstly, exposure to anti-conspiracy arguments both before and after exposure to conspiracy theories can restore vaccination intention (Jolley and Douglas, 2017; Lyons et al., 2019) As for Chen et al. (2020), they observed that pre-existing knowledge about the HPV vaccine nullified the impact of exposure to anti-vaccines conspiracy theories on HPV vaccination intention. Altogether, these results emphasize the relevance of both proactive information and misinformation correction initiatives before the public is exposed to misinformation, and debunking efforts after exposure, to reduce the impact of COVID-19 conspiracy beliefs.

In conclusion, our results are congruent with past research and suggest that when a vaccine against COVID-19 becomes available, conspiracy beliefs of all kinds might slow down the population’s immunization. This should encourage academics, policy makers, health authorities, and journalists to start working on initiatives to tackle this issue.

## Author’s Note

By the time this manuscript was accepted, talking about “hydroxychloroquine” had become more common than talking about “chloroquine”. However we chose to use the latter in the text which we believe improve the readability.

## Data Availability Statement

The datasets presented in this study can be found in online repositories. The names of the repository/repositories and accession number(s) can be found in the article/Supplementary Material.

## Ethics Statement

The university of the leading authors does not have institutional review boards for psychology or social science research. We thus applied the 1964 Helsinki Declaration and its later amendments (2001), the ethical principles of the French Code of Ethics for Psychologists (2012), and the American Psychological Association Ethical Principles of Psychologists and Code of Conduct (2017). Participants were informed about the purpose of the study in a cover letter and were assured that their data would remain confidential. Participants had to give explicit written consent to access the study.

## Author Contributions

PB conceptualized this research project, contributed to the methodology development, data collection, data curation, statistical analysis, original draft, and did the project administration and supervision. KN contributed to the methodology, data curation, statistical analysis, and original draft. SD contributed to the methodology development, data collection, and original draft. All authors contributed to the article and approved the submitted version.

## Conflict of Interest

The authors declare that the research was conducted in the absence of any commercial or financial relationships that could be construed as a potential conflict of interest.

## Footnotes

1 https://osf.io/x43na/?view_only=53Lp3XwFS4e7GGH8WseizHm1pd6nkJmqcz2
