# Supplementary material for: Conspiracy Beliefs, Rejection of Vaccination, and Support for hydroxychloroquine: A Conceptual Replication-Extension in the COVID-19 Pandemic Context
Source: Front Psychol. 2020 Sep 18;11:565128. doi: 10.3389/fpsyg.2020.565128 (PMC7536556; doi:10.3389/fpsyg.2020.565128)
Supplement: Supplementary file 1 [file Table_1.pdf]

Supplementary Analysis

Study 1

Table 1.

Geographic distribution for Study 1

Locality	*N*
Auvergne-Rhône-Alpes	20 (4.8%)
Bourgogne-France-Comté	1 (0.2%)
Bretagne	52 (12.7%)
Centre-Val de Loire	3 (0.7%)
Corse	3 (0.7%)
Grand Est	4 (0.9%)
Hauts-de-France	4 (0.9%)
Îles-de-France	30 (7.3%)
Normandie	16 (3.9%)
Nouvelle-Aquitaine	9 (2.2%)
Occitanie	7 (1.7%)
Pays de la Loire	17 (4.1%)
Provence-Alpes-Côte d’Azur	231 (57.9%)
Régions d’Outre-mer	3 (0.7%)
Other	9 (2.2%)

Table 2.

Factor loadings for exploratory Factor Analyses of COVID-19 conspiracy beliefs

	Factor 1	Factor 2
1. Chinese bioweapon	.84	
2. Chinese economic crisis	.88	
3. Chinese failure	.76	
4. Hidden vaccine	.58	
5. Industrialist strategy		.47
6. Eugenics motives	.77	
7. French pension reform		.63
8. French government laxity		.68
9. French municipal election		.81

Note. Using Oblimin rotation.

Study 2

Table 3.

Distribution of the education

High school	College	Bachelor's degree	Master's degree	Doctoral degree	Other	Missing
0.7 % (N = 3)	14.6 % (N = 58)	58.3 % (N = 231)	15.9% (N = 63)	2.2 % (N = 9)	2.7 % (N = 11)	5.3 % (N = 21)

Table 4.

Factor loadings for exploratory Factor Analyses of COVID-19 and chloroquine conspiracy beliefs

	**Factor 1**	**Factor 2**	**Factor 3**
1. Chinese bioweapon	0.933		
2. Chinese economic crisis	0.938		
3. Chinese failure	0.587		
4. Hidden vaccine	0.556		
5. Industrialist strategy			0.495
6. Eugenics motives	0.734		
7. Pasteur Institute	0.506		
8. French pension reform			0.758
9. French government laxity			0.794
10. French municipal election			0.639
11. Chloroquine financial conflict		0.859	
12. Chloroquine media bashing		0.785	
13. Chloroquine government		0.583	
14. Chloroquine prescription		0.722	
15. Pharmaceutical industry and scientists		0.818	

Note. Using Oblimin rotation.
